# Fast and Sensitive Ellipsometry-Based Biosensing

**DOI:** 10.3390/s18010015

**Published:** 2017-12-22

**Authors:** Kewu Li, Shuang Wang, Liming Wang, Hui Yu, Ning Jing, Rui Xue, Zhibin Wang

**Affiliations:** 1 School of Information and Communication Engineering, North University of China, Taiyuan 030051, China; kewuli1990@gmail.com (K.L.); wlm@nuc.edu.cn (L.W.); jingning@nuc.edu.cn (N.J.); 2 Engineering Technology Research Center of Shanxi Province for Opto-Electric Information and Instrument, Taiyuan 030051, China; S1507038@st.nuc.edu.cn (S.W.); 13934603474@nuc.edu.cn (H.Y.); xuerui@nuc.edu.cn (R.X.)

**Keywords:** biosensing, ellipsometry, photoelastic modulator, biolayer, refractive index, effective thickness

## Abstract

In this work, a biosensing method based on in situ, fast, and sensitive measurements of ellipsometric parameters (*Ψ*, ∆) is proposed. Bare silicon wafer substrate is functionalized and used to bind biomolecules in the solution. Coupled with a 45° dual-drive symmetric photoelastic modulator-based ellipsometry, the parameters *Ψ* and ∆ of biolayer arising due to biomolecular interactions are determined directly, and the refractive index (RI) of the solution and the effective thickness and surface mass density of the biolayer for various interaction time can be further monitored simultaneously. To illustrate the performance of the biosensing method, immunosensing for immunoglobulin G (IgG) was taken as a case study. The experiment results show that the biosensor response of the limit of detection for IgG is 15 ng/mL, and the data collection time is in milliseconds. Moreover, the method demonstrates a good specificity. Such technique is a promising candidate in developing a novel sensor which can realize fast and sensitive, label-free, easy operation, and cost-effective biosensing.

## 1. Introduction

To monitor biorecognition and binding events is crucial in the healthcare, pharmaceutical, and biotechnology fields. Most metabolic and immunological effects are followed from the interactions between biomolecules like antibodies, DNA, RNA, proteins, or whole cells [1]. The information on the binding of a ligand to its receptor can provide instructions in the drug discovery process, and the accurate detection of disease biomarker has been widely applied for disease diagnosis and state monitoring [2,3]. In the past few decades, various sensor-based platforms have been employed to determine the specificity, kinetics, and affinity of a wide variety of biomolecular interactions and the concentration of analyte [4,5,6,7], while the search for new method is of constant interest and challenge in this scientific field in order to develop more robust, rapid, sensitive, and cost-effective biosensing platforms.

Radioimmunoassay and fluorescence sensors are sensitive, but some types of radiolabelling or fluorescent labelling are required [8,9]. This more or less interferes with the molecular interaction by occluding the binding sites, and the inconsistent yields of target synthesis and labeling as well as the nonuniform rates of fluorophore photobleaching can decrease the readout accuracy. This labeling step imposes extra time, and the cost and sophistication of the instrumentation are increased. An electrochemical biosensor directly emits electronic signals due to changes in the electronic properties of the electrode surface introduced by biointeractions, which offers label-free and rapid detection [10]. However, the fabrication processes of the high sensitive electrochemical sensor devices equipped with biologically compatible electrode are complex and expensive. Interferometric optical biosensor (e.g., Mach-Zehnder interferometer, Young interferometer, Hartman interferometer, etc.) can measure binding interactions in free solution while using small amounts of sample and maintaining a straightforward and inexpensive format, but the sensor is affected by environment easily, especially the ambient vibration and the temperature variation [11]. Surface plasmon resonance (SPR) biosensor applies the evanescent waves to probe the small changes in the refractive index (RI) near the thin metal film substrate, and the interactions between immobilized receptors and analytes in the solution are analyzed [12]. The SPR technique has become a research hotspot in biosensing field, and has gradually been commercialized. Most of current commercial SPR sensors are amplitude-sensitive interrogations based on the control of the position of reflectivity dip in angular or wavelength spectra, or the intensity under a fixed angle of incidence and wavelength. To further improve the sensitivity, interferometry or phase modulation methods are also used to determine the phase jump near the zero-reflection point, the detection limit of such phase-sensitive approach can be of the order of 10^−8^ RI units (RIU) [13,14,15]. It is, however, inescapable that the fluctuations of RI that would lead to false positive signals and inevitable noise [16]. These thorny problems restrict the implementation and use of the SPR method in the detection of biomolecules with low concentration and low molecular-weight.

Ellipsometry, known as the best technique for surface studying, is also reported to be directly used to determine the biolayer, and various strategies, such as imaging ellipsometry and spectroscopic ellipsometry, are employed for biosensing applications [17,18,19]. It has become a practical technique for protein detection with functions of in situ, label-free, non-destructive, sensitive characterization for protein interaction process and quantification, and high-throughput measurements can be realized using imaging ellipsometry [20,21,22]. However, most of these ellipsometers are the configuration of Polarizer-Compensator-Specimen-Analyzer (PCSA) or null ellipsometry, it needs to rotate the optical element (polarizer or compensator) when the ellipsometer works. The frequency of the mechanical rotating analyzer or compensator (typically tens of hertz) is slow, which limits the sensing speed. Also, the artifacts such as the system instability and light beam drift caused by the mechanical rotation are difficult to eliminate. Although some imaging ellipsometry works in off-null ellipsometry mode [23,24], the sensing speed is limited by the frame rate of CCD camera (typically tens of fps), and sensing sensitivity is effected by the fluctuation and distribution uniformity of light beam. In addition, for large numbers of ellipsometry biosensing applications, the analytes must first be bound to either the silicon (Si) substrate with several hundred nanometers silica film or glass substrate with several nanometers gold film, then the unbound biomolecules are rinsed off, and the film is dried before performing ellipsometric measurements. Obviously, this cannot be applied to study the biointeraction in the solution environment and real-time, and the sensitivity is also limited. The method of SPR Ellipsometry (SPRE) combining spectroscopic ellipsometry and the Kretschmann type SPR geometry of total internal reflection is also proposed [25]. The detection of analytes at very low concentration down to 1 pM can be obtained, but the problems of low sensing speed and SPR noises still need to be solved.

We recently demonstrated a 45° dual-drive symmetric photoelastic modulator (PEM) [26,27]. An in situ ellipsometry using the 45° dual-drive symmetric PEM was built, and fast, sensitive, and full range measurements of the ellipsomtric parameters (*Ψ*, ∆) were realized [28]. In this work, we apply the 45° dual-drive symmetric PEM-based ellipsomtry to determine the parameters *Ψ* and ∆ of the biolayer formed as a result of the biomolecular interactions in the solution. Bare Si wafer substrate is functionalized and used to bind biomolecules. The simultaneous measurements of *Ψ* and ∆ provide rich information about the analyte in liquid media under investigation. The RI of the solution and the effective thickness and surface mass density of the biolayer for various interaction time can be found out further. Thus, a novel ellipsometry-based sensor is expected to be developed, and highly accurate and sensitive, label-free, in situ, real-time, easy operation and cost-effective biosensing can be achieved.

## 2. Methods

### 2.1. Optical Model and Numerical Calculation

**Figure 1:**
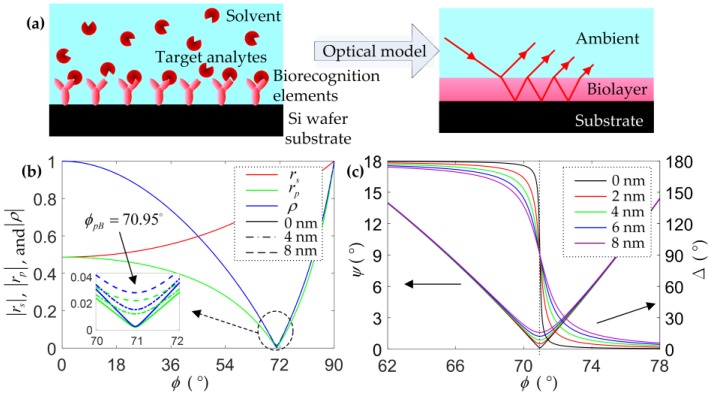
Optical model and numerical calculation. (**a**) solution ambient–biolayer–substrate model; (**b**) the amplitudes of *r_p_*, *r_s_*, and *r*; (**c**) the ellipsometric parameters (*Ψ*, ∆) at different thicknesses of the SiO_2_ thin film varies with the incident angle (*φ*). |*r_p_*|, |*r_s_*|, and |*r*| represent the amplitudes. *φ_pB_* is the pseudo-Brewster angle for parallel (p)-polarized light.

The biomolecular interactions between the target analytes and biorecognition elements binding to the substrate can be regarded as a three-layer optical model, solution ambient–biolayer–substrate, as shown in Figure 1a. The Fresnel reflection coefficients used to describe the reflection of light at each interface, *r_p_* and *r_s_* for the parallel (p) and perpendicularly (s) polarized light components [29], respectively, are given by

(1) $$\{\begin{array}{c} r_{pij}=\frac{N_{j}\cos\phi_{i}-N_{i}\cos\phi_{j}}{N_{j}\cos\phi_{i}+N_{i}\cos\phi_{j}}\\ r_{sij}=\frac{N_{i}\cos\phi_{i}-N_{j}\cos\phi_{j}}{N_{i}\cos\phi_{i}+N_{j}\cos\phi_{j}} \end{array}.$$

Similarly, the transmission *t_p_* and *t_s_* coefficients can be described as

(2) $$\{\begin{array}{c} t_{pij}=\frac{2N_{i}\cos\phi_{j}}{N_{j}\cos\phi_{i}+N_{i}\cos\phi_{j}}\\ t_{sij}=\frac{2N_{j}\cos\phi_{j}}{N_{i}\cos\phi_{i}+N_{j}\cos\phi_{j}} \end{array}$$

where, *N_i_* and *N_j_* are the refractive indices of the media on both sides of the interface. *φ_i_* and *φ_j_* are the angles of incidence and refraction, respectively, and they satisfy the Snell’s law (*N_i_* sin*φ_i_* = *N_j_* sin*φ_j_*). The ratio of *r_p_* to *r_s_* defines the ellipsometric parameters as

(3) $$\rho =\frac{r_{p}}{r_{s}}=\tan\psi e^{i\Delta }$$

where, $\psi =\tan^{-1}(\left|\rho \right|)$ is amplitude ratio, and $\Delta =\delta_{p}-\delta_{s}$ is phase difference. In order to predict and optimize the biosensing performance of the ellipsometry measurement approach, the analysis of numerical calculation for the three-layer system is carried out, as shown in Figure 1b,c. The ellipsometric parameters (*Ψ*, ∆) for increasing the thickness of a SiO_2_ thin film on the Si (100) wafer substrate immersed in de-ionized (DI) water is simulated. At the incident light with a wavelength of 650 nm, the RI of the DI water is 1.33, the optical constants of the SiO_2_ thin film are similar to that of the biolayer (average thickness of 3–5 nm), the RI is about 1.4565 [30,31], and the complex RI of the Si wafer is 3.8515–0.01646i [32]. The amplitudes of *r_p_*, *r_s_*, and *r* and the parameters *Ψ* and ∆ at different thicknesses of the SiO_2_ thin film are dependent on the incident angle (*φ*) of the light beam.

**Figure 2:**
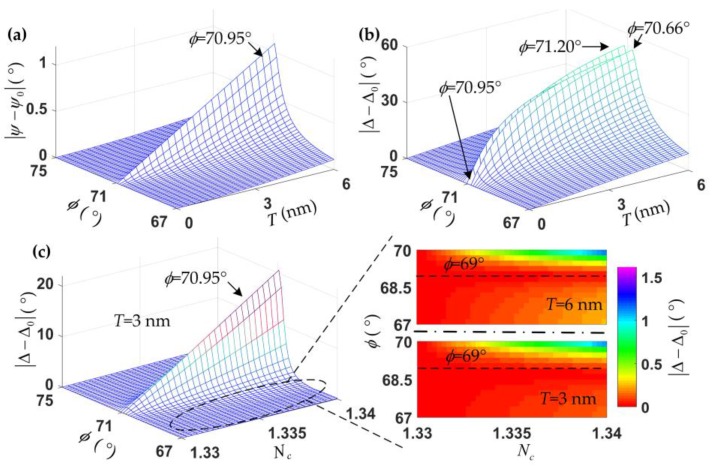
*Ψ* (**a**) and ∆ (**b**) change with the incident angle (*φ*) and the film thickness (*T*). |*Ψ* − *Ψ*_0_| and |∆ − ∆_0_| represent the changes in *Ψ* and ∆, respectively. (**c**) ∆ changes with the incident angle (*φ*) and the refractive index (RI) of the ambient solution (*N_c_*). Where (*Ψ*_0_, ∆_0_) represent the initial values of the two ellipsometric parameters; in Figure 2a,b, *Ψ*_0_ and ∆_0_ are for different *φ* from 67° to 75° when *T* is 0 nm; in Figure 2c, ∆_0_ is for different *φ* from 67° to 75° when *N_c_* is 1.33 and *T* is 3 nm.

The curves of the amplitudes of *r_p_*, *r_s_*, and *ρ* at different thin film thicknesses over the incident angle nearly coincide. When the incident angle is 70.95°, the amplitudes of *r_p_* and *ρ* reach the minimum values; this angle is the so-called pseudo-Brewster angle [33]. As shown in Figure 1c, *Ψ* varies rapidly with the film thickness at the pseudo-Brewster angle, while ∆ keeps a constant. However, ∆ changes larger than *Ψ* with the film thickness nearby the pseudo-Brewster angle, and the thinner the film thickness is, the rapider the ∆ changes occur. This is further illustrated in Figure 2. In the numerical calculation, *T* represents the thickness of the thin film, the range is set at 0 to 6 nm, and *N_c_* represents the RI of the ambient solution; the range is set at 1.33 to 1.34.

Figure 2a,b further show that ∆ is much more sensitive than *Ψ* to the changes in film thickness, while ∆ does not change at the pseudo-Brewster angle, and *Ψ* is reversed. When the angle of incidence is 70.66° or 71.20°, the maximal film thickness change rate of ∆ is achieved, but the maximal change rate almost has the worst linearity. The dynamic range of the ambient solution RI is assumed to be from 1.33 to 1.34, the changes in ∆ with the RI at different angles of incidence is calculated, and the results are shown in Figure 2c, the film thickness being 3 nm. The parameter ∆ is significantly changed with the RI of the ambient solution when the angle of incidence is the pseudo-Brewster angle. However, there is almost no change in ∆ with the ambient solution RI for different film thicknesses, when the incident angle is near the pseudo-Brewster angle, especially at the angle of about 69°. Therefore, bare Si wafer is used as the substrate, and the angle of incidence is chosen nearby the pseudo-Brewster angle, it is expected to achieve an ellipsometry-based biosensing method which is sensitive to the thin film thickness growth but independent of the changes in the solution RI.

### 2.2. Biosensing Scheme

**Figure 3:**
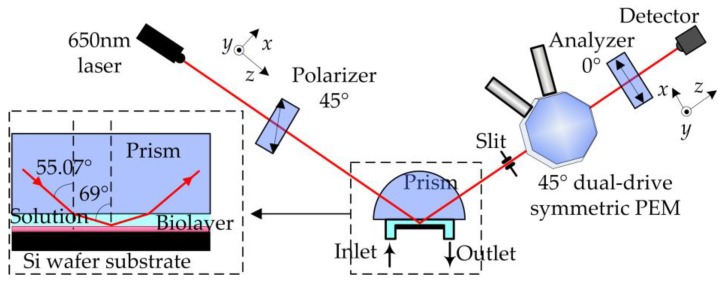
Schematic of the biosensing platform. A micro-fluidic sensor cell is built based on a semicylindrical prism, the prism material is BK7, and the RI is 1.5145 for the incident light with a wavelength of 650 nm. The structure of the sensor cell chip is shown in the inset. A slit is used to eliminate the interferences from the light beam that is directly reflected by the bottom surface of the prism. The ellipsometric parameters of the biolayer are measured by using a 45° dual-drive symmetric photoelastic modulator (PEM).

In order to measure the ellipsometric parameters of the biolayer immersed in the solution sensitively and precisely, two key problems should be solved. The angle of incidence of light beam to the biolayer should be set at near the pseudo-Brewster angle (about 69°), and the ellipsometric system should be sensitive enough to measure the parameters *Ψ* at near 0° and ∆ at near 180°. The biosensing platform was carefully designed, as depicted in Figure 3.

The platform mainly consists of two parts (Figure 3), the sensor cell and the ellipsometric parameter measurement apparatus. It is known that the angle of incidence should not exceed the critical angle of the air-solution interface (about 48.75°) if the light is directly propagated from air to the sensor chip after passing through the solution. Therefore, a coupled semicylindrical prism is introduced. The prism material is BK7, and the RI is 1.5145 for the incident laser light with a wavelength of 650 nm. According to Snell’s law, the incident angle to the biolayer is set at near pseudo-Brewster angle 69° when the angle of incidence in prism is 55.07°. A micro-fluidic sensor cell is also built based on the prism. The solution under investigation is injected from the inlet, then the target analytes interact with the biorecognition elements binding to the bare Si wafer substrate, and finally, the waste is flowed from the outlet.

To rapidly and sensitively monitor the specificity, kinetics, and affinity of the biomolecular interactions and the concentration of the analyte by determining the changes in the biolayer system, the method of in situ measurements of the ellipsometric parameters using a 45° dual-drive symmetric PEM is employed. As reported in detail, the 45° dual-drive symmetric PEM works in the pure traveling-wave modulation mode [28], the retardation magnitude of the PEM is set to π/2, and the modulation axis performs circular motion at the half of the PEM resonance frequency. The PEM operation control and data processing are based on a field programmable gate array (FPGA). The in situ, fast, sensitive, and full range measurements of *Ψ* and ∆ are realized. Comparing to rotating-analyzer ellipsometry (RAE) and rotating-compensator ellipsometry (RCE), this method has the following advantages: full range and uniformly sensitive measurements of *Ψ* and ∆ can be obtained, the artifacts such as the system instability and light beam drift (for RAE and RCE) caused by the mechanical rotation are eliminated, and the PEM with typical working frequency of 50 kHz are much larger than that of the RAE or RCE about several or tens of Hertz, which lead to achieving accurate, sensitive and fast measurements.

**Figure 4:**
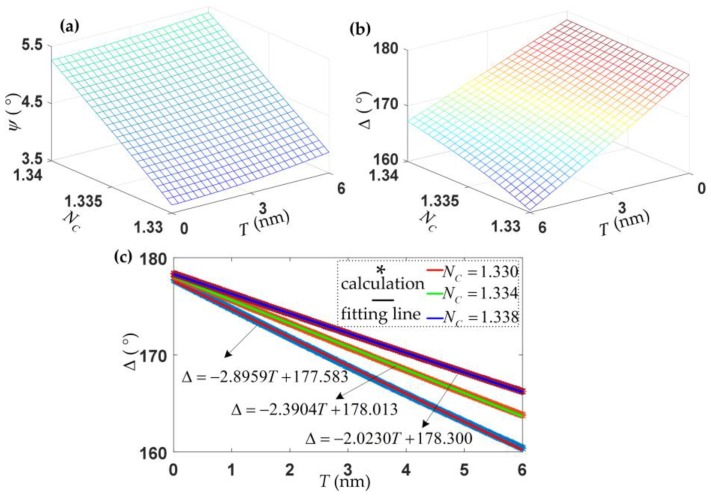
Numerical calculations of the parameters *Ψ* and ∆. *Ψ* (**a**) and ∆ (**b**) change with the film thickness *T* and the RI of the solution *N_C_*. The angle of incidence in the prism is 55.07°, the angle of incidence to the biolayer will change from 69° to 67.90° with the changes of *N_C_* from 1.33 to 1.34, which results in different slopes (**c**) of ∆ at the biolayer thicknesses *T*.

However, according to the Snell’s law, the angle of incidence to the biolayer system (the sensor cell shown in Figure 3) will change with the RI of the ambient solution when the incident angle in the prism is set. This is worth noting for in situ, fast, or even real-time biosensing. For the entire sensor cell platform, the ratio of *r_p_* to *r_s_* can be re-expressed as

(4) $$\rho^{\prime }=\frac{t_{pPC}r_{pCBS}t_{pCP}}{t_{sPC}r_{sCBS}t_{sCP}}$$

where *P*, *C*, *B*, and *S* represent the prism, solution, biolayer, and bare Si wafer substrate, respectively. The angle of incidence in the prism is set at 55.07°, the numerical calculations of the parameters *Ψ* and ∆ are shown in Figure 4.

According to Figure 2a and Figure 4a, the changes in *Ψ* with *N_C_* are almost linear, and the changes resulting from the changes of the film thicknesses are much smaller. According to Figure 4b,c, ∆ shows a linear correlation with the film thickness, but the slopes vary for different incidence angles due to different *N_C_*. In Equation (5), the curve of *Ψ* versus *N_C_* and the plane curve of ∆ are both fitted.

(5) $$\{\begin{array}{c} N_{C}=0.0062\psi +1.306999\\ \begin{array}{l} \Delta =-[(0.38833872{N_{C}}^{2}-1.04696904N_{C}+0.70582545)T\\ \;+(0.38835009N_{C}{}^{2}-1.04502775N_{C}+0.68517524)]\times 10^{4} \end{array} \end{array}$$

The simultaneous measurements of *Ψ* and ∆ provide rich information about the analytes under investigation. According to Equation (5), the RI of solution and the thickness of the biolayer can be measured at the same time.

## 3. Experiments

In order to verify and apprise the performance of the ellipsometry-based biosensing method, the immunosensing for immunoglobulin G (IgG) is investigated as a case study.

### 3.1. Materials

Si (100) wafers were purchased from LIJINGKEJI, Ltd., Quzhou, China. 3-aminopropyltriethoxysilane (APTES), glutaraldehyde, phosphate buffered saline (PBS, pH7.4), and ethanolamine hydrochloride were purchased from Shanghai Macklin Biochemical Co., Ltd. (Shanghai, China). Human immunoglobulin G (IgG), mouse IgG, rabbit IgG and goat anti-human IgG were purchased from Beijing Solarbio Science & Technology Co., Ltd. (Beijing, China). De-ionized (DI) water was used during the entire experiment, and all other chemicals were of analytical reagent grade.

### 3.2. Bare Si Substrate Functionalized and Sensor Cell Fabricated

A 15 × 15 mm Si (100) wafer is used as the substrate. Before the biosensing determination, these functionalization processes of the substrate are essential. First, the Si substrates were cleaned by hydrofluoric acid for about 2 min to remove native SiO_2_ layer. Then, the bare Si substrates were further cleaned in an ultrasound bath with acetone for 10 min and with ethanol for another 10 min, and finally with DI water for 20 min. Thereafter, the bare Si substrates were hydroxylated in freshly prepared Piranha solution (70% H_2_SO_4_–30% H_2_O_2_) for 30 min, rinsed with a copious amount of DI water, and dried in a stream of nitrogen gas. Next, the Si substrates were incubated in ethanol solutions of APTES with a concentration of 5.0% (*v*/*v*) for 1 h to aminate the substrates. After the controlled deposition, the Si substrates were sonicated twice in ethanol for 10 min to remove loosely physisorbed APTES. These substrates were then dried under nitrogen gas.

To carry out the biosensing in liquid media, a micro-fluidic flow sensor cell was fabricated using the Si substrate coupled with a semicylindrical prism. A thin polydimethylsiloxane (PDMS) pad of about 1 mm thickness was used to support and seal the edges of the Si substrate to the bottom surface of the prism, and a center chamber was built to hold the solutions. The PDMS also helps to make two micro channels that allow the solution to be injected from the inlet and the waste to be flowed through the outlet. In the biomolecular interaction study, PBS was first injected to wash the sensor cell, then the PBS solution of glutaraldehyde with a concentration of 1.5 wt % was injected into the flow cell for 2 h to make the Si substrate aldehydated, and glutaraldehyde plays as a crosslinking agent to immobilize the antibody to the surface of the Si substrate. Finally, PBS was flowed to wash the unreacted glutaraldehyde solution. Thereafter, 125 µg/mL of goat anti-human IgG was injected into the sensor cell for 12 h to reach saturation. Before immunosensing, 1 M ethanolamine hydrochloride (pH 8.0) was used to block the non-specific binding sites. The sensor cell with the functionalized substrate thus got ready to measure the interaction between antibody and antigen.

### 3.3. Ellipsometry Apparatus

The ellipsometric parameter signals with different biointeraction times were investigated using our homemade 45° dual-drive symmetric PEM-based ellipsometry. A low noise laser diode operating at the wavelength of 650 nm and output power of 5 mW was employed as the light source. Both the polarizer and analyzer were Glan–Taylor polarizers with an extinction ratio greater than 10^5^:1. The working frequency of the 45° dual-drive symmetric PEM is 49.956 kHz. An Altera EP3C FPGA was used to provide the PEM driving signals, as well as control a fast and precise 12 bit analog-to-digital converter (ADC) clock frequency, and finally complete the digital signal processing. The sampling frequency of the ADC was adjusted to 3.2 MHz, and 50 integer periods of the PEM, about 1 ms was chosen for one single data digital processing output.

## 4. Results and Discussion

### 4.1. Baseline Determination

**Figure 5:**
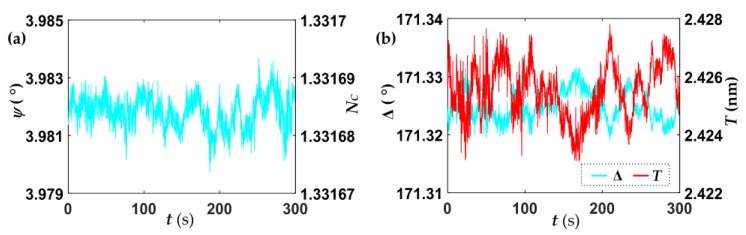
Ellipsometric parameters of *Ψ* (**a**) and ∆ (**b**) measured under constant flow of phosphate buffered saline (PBS) buffer, the RI of solution *N_C_* and the thickness of the biolayer *T* were solved from Equation (5).

First, PBS buffer was injected into the sensor cell until the ellipsometric parameters signals became and kept constant. The baseline was determined, as shown in Figure 5

Each output data value is the average of one hundred measurements, so the acquiring time is adjusted as 100 ms/data. In combination with Equation (5), the RI of solution *N_C_* and the thickness of the biolayer *T* can be monitored with the measurements of ellipsometric parameters *Ψ* and ∆, and the parameters of the baseline are recorded in Table 1.

The RI of PBS is 1.331687, and the thickness of the biolayer, after covalently binding the anti-IgG to the bare Si wafer substrate, is 2.425 nm, which represent the initial values of the biosensing platform. The standard deviations of the ellipsometric parameters are both at the order of 10^−30^, which correspond to the standard deviations of the solution RI and biolayer thickness are 6 × 10^−6^ and 1 pm, respectively. This also indicates that the fine repeatability and sensitivity of the platform are obtained, and the solution RI measurement sensitivity of our biosensing platform are on a par with that of SPR sensors based on angular or wavelength interrogation [12], while our biosensor achieves a larger dynamic range.

### 4.2. Biointeraction Observation

**Figure 6:**
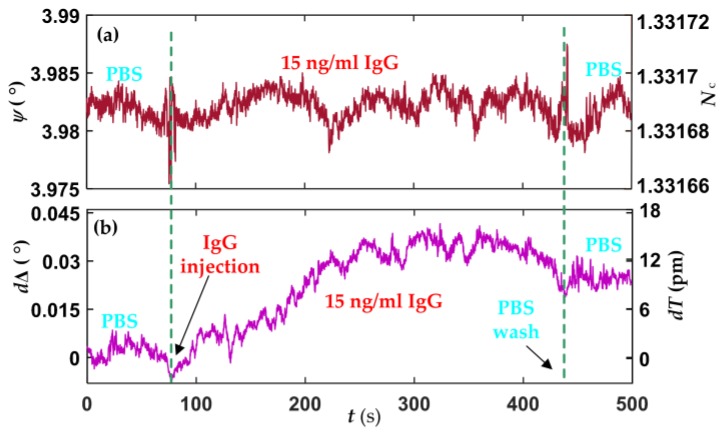
Limit of detection (LOD) of human IgG, (**a**) *Ψ* and (**b**) ∆ signals.

Human IgG in the concentration range of 15–1000 ng/mL in PBS were flowed over the sensor cell, followed by washing with PBS after every injection. Figure 6 shows the platform response of the limit of detection (LOD) of human IgG.

**Figure 7:**
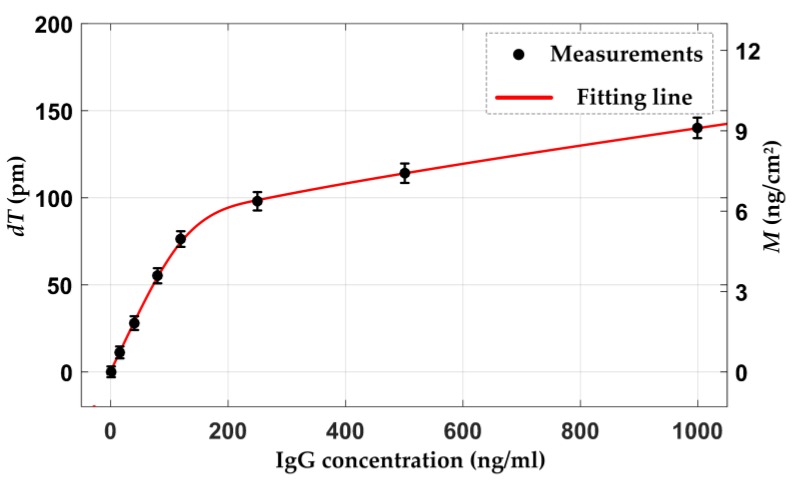
Concentration-dependent response.

There are no obvious changes in *Ψ* signal after human IgG being injected, as shown in Figure 6a, suggesting the RI of 15 ng/mL human IgG in PBS is similar to the RI of PBS. Figure 6b shows changes in ∆ (*d*∆) signal, which unveils the interactions between IgG and anti-IgG. After the biointeractions had sustained for about 6 min, PBS was injected to wash out the unbound IgG. Using Equation (5), the growth of the biolayer thickness (*dT*), about 11 pm, can be observed accordingly. In fact, *dT* corresponds to the effective thickness of human IgG, and the surface mass density of human IgG can also be evaluated by using de Feijter’s equation [34].

(6) $$M=dT\frac{N_{B}-N_{C}}{dn/dc}$$

where *N_B_* is the RI of the biolayer. *dn*/*dc* is the RI increment of the human IgG solution, and the value is taken as 0.190 mL/g with IgG molecular mass about 150 kDa. The LOD of 15 ng/mL human IgG corresponds the surface mass density of 0.72 ng/cm^2^. The responses of our platform for the other concentrations human IgG were also performed and shown in Figure 7.

The biolayer thickness and surface mass density rapidly increase at the lower concentration range, and the trend slows down at the higher concentration range, which reveals that the functional antibody surface provides available active binding sites at the start and gradually reaches saturation with the increase of the human IgG concentration. In addition, there are no changes in the parameter *Ψ* for all concentrations human IgG immunosensing experiments, and the RI of the solution nearly remains constant at 1.331687. In fact, the RI increment of the IgG solution is 0.190 mL/g [35], which suggests the concentration range change from 15 to 1000 ng/mL corresponding to the changes in RI are no more than 1.9 × 10^−7^, this beyond the sensitivity, 6 × 10^−6^, of our biosensing platform. However, the differences in the RIs of different solvents, such as DI water, PBS, and serum, are significant. It is essential to detect the RI baseline of the solution precisely and guarantee the in situ measurement for our biosensing platform, and any abnormal environment or instrumentation signal can also be monitored and eliminated by real-time measurement of the solution RI.

For comparison, analytical performance of several reported biosensing methods were listed in Table 2.

The detection limit of our platform is comparable with that of the other method, particularly those based on SPR and electrochemistry techniques, while the high measurement sensitivity of our method is achieved without using any noble metal nanoparticles amplification strategy. So, our method is label-free and cost-effective. Our method also has a faster response than the other reported methods, as the data collection time of our platform can be up to 1 ms. This has good prospects for the analysis and application of rapid biointeraction process.

### 4.3. Specificity Evaluation

**Figure 8:**
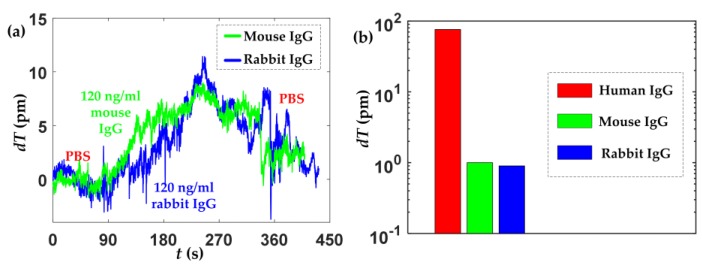
Specificity evaluation, (**a**) the biosensing responses of mouse IgG and rabbit IgG, (**b**) changes in the effective thicknesses of the biolayers for different IgG (human IgG, mouse IgG, and rabbit IgG).

The evaluation of the specificity was also performed by detecting mouse IgG and rabbit IgG. Two new Si wafer substrates were functionalized with anti-human IgG film, as the procedures described in Section 3.2. Mouse IgG and rabbit IgG with the concentration of 120 ng/mL in PBS was separately incubated in the micro-fluidic sensor cell for about 6 min, then PBS was injected to rinse the sensing film. There are almost no changes in the effective thicknesses of the biolayers on the two Si substrates, as shown in Figure 8.

Figure 8a shows that the thickness slightly increases upon injection of mouse and rabbit IgGs and decrease after washed by PBS, the origin of the increase of thickness is due to nonspecific adsorption. Thus, the results indicate that the fabricated substrates with anti-human IgG in this work has the ability of selective detection of human IgG. The biosensor has a good specificity performance. For different biomolecules detection, it only needs to replace and functionalize the bare Si wafer substrate with target biorecognition element and seal the substrate to the sensor cell, thus making the biosensing method also easy to operate.

## 5. Conclusions

We have presented a complete biosensing platform that comprises a 45° dual-drive symmetric PEM-based ellipsometry and a sensor cell constructed by a bare Si wafer substrate and a semicylindrical prism. With the in-situ, fast and sensitive measurements of the ellipsomtric parameters, the RI of the solution and the effective thickness and surface mass density of the biolayer for various interaction time are monitored. The experiment results showed that the standard deviations of *Ψ* and ∆ are both at the order of 10^−30^, which correspond to the repeatability and sensitivity of the solution RI and biolayer thickness are 6 × 10^−6^ and 1 pm, respectively. Compared with the other biosensing methods, particularly those based on SPR and electrochemistry, the high measurement sensitivity of our method is achieved without using any noble metal nanoparticles amplification, and our method has realized faster response, as the data collection time can be up to 1 ms. Therefore, our ellipsometry-based biosensing method is a promising candidate in developing a novel sensor which can provide the simultaneous measurement of the RI of solution and the thickness and surface mass density of the biolayer, rendering it suitable for highly accurate and sensitive, in situ, real-time, label-free, easy operation and cost-effective biosensing.

## Figures and Tables

**Table 1:** Parameters of baseline.

Parameters	Mean Value	Standard Deviation
*Ψ* (°)	3.982	0.001
∆ (°)	171.325	0.003
*N_C_*	1.331687	6 × 10^−6^
*T* (nm)	2.425	0.001

**Table 2:** Comparison with reported biosensing methods.

Method	Measurement Strategy	Analyte	Detection Limit	Response Time Level	References
SPR	AgNCs ^1^ + chitosan	Mouse IgG	0.6 µg/mL	<1 min	[36]
	Petide SAM ^2^	Human IgG	0.45 ng/mL (3 pM)	<1 min	[37]
	PSPW ^3^	Mouse IgG	10 pg/mL	<1 s	[15]
Fluorescence	Fluorescence microsope	Horse IgG	0.71 µg/mL	25 min	[38]
Electrochemistry	CAuNCs ^4^	Rabbit IgG	5 ng/mL	<1 s	[39]
	ELISA ^5^	Goat IgG	1 ng/mL	2–5 min	[40]
Ellipsometry	RCE ^6^ + Porous silicon	Albumin	-	<10 s	[41]
	Imaging	AFP ^7^	5 ng/mL	<1 s	[22]
SPRE	SPR + Ellipsometry	β-Cyclodextrins	1 pg/mL (1 pM)	2 s	[25]
Our platform	45° dual-drive symmetric PEM + Bare Si	Human IgG	15 ng/mL	1 ms	Present work

^1^ AgNCs: Ag nanocubes; ^2^ SAM: self-assembled monolayer; ^3^ PSPW: paired surface plasma wave; ^4^ CAuNCs: concave gold nanocuboids; ^5^ ELISA: enzyme linked immunosorbent assay; ^6^ RCE: rotating-compensator ellipsometry; ^7^ AFP: alpha-fetoprotein.
